# Acinar-ductal metaplasia in pancreatitis and pancreatic ductal adenocarcinoma

**DOI:** 10.1007/s13402-026-01251-0

**Published:** 2026-06-29

**Authors:** Xiangyu Zhang, Yutong Zhao, Cheng Qin, Zeru Li, Yiping Xie, Yutong Yan, Tianyu Li, Lirui Huang, Haoyu Shi, Weibin Wang

**Affiliations:** 1 Peking Union Medical College Hospital, Department of General Surgery, Peking Union Medical College, Chinese Academy of Medical Sciences, Beijing, 100023 P.R. China; 2 Key Laboratory of Research in Pancreatic Tumor, Chinese Academy of Medical Sciences, Beijing, 100023 P.R. China; 3 National Science and Technology Key Infrastructure on Translational Medicine in Peking Union Medical College Hospital, Beijing, 100023 P.R. China

**Keywords:** Pancreatic ductal adenocarcinoma, Acinar-ductal metaplasia, Pancreatitis, Preneoplastic intraepithelial neoplasia

## Abstract

Acinar-ductal metaplasia (ADM) represents a dynamic and reversible cellular adaptation in the pancreas, whereby acinar cells dedifferentiate and transdifferentiate into duct-like cells in response to inflammatory, metabolic, and environmental stressors. While transient ADM facilitates tissue repair after acute injury, persistent stimuli—including chronic pancreatitis, metabolic dysregulation, obesity, diabetes, smoking, and oncogenic KRAS mutations—stabilize ADM and promote progression to pancreatic intraepithelial neoplasia (PanIN) and pancreatic ductal adenocarcinoma (PDAC). ADM is orchestrated by integrated signaling networks involving TGF-β/SMAD, EGFR–KRAS–MAPK, PI3K–AKT–mTOR, Notch, Wnt/β-catenin, and JAK/STAT3, which regulate dedifferentiation, proliferation, survival, and fibrosis. Inflammatory cells, particularly macrophages and eosinophils, and pro-inflammatory cytokines such as IL‑6, IL‑18, and TNF‑α further amplify ADM and create a tumor-promoting microenvironment. Single-cell analyses reveal that ADM encompasses heterogeneous cell states with distinct lineage trajectories, determining reversibility or pre-neoplastic transformation. Pharmacological interventions, including HDAC inhibitors, JAK/STAT3 inhibitors, metformin, and anti-inflammatory agents, show potential in preventing or reversing ADM. Understanding the complex molecular, cellular, and environmental regulation of ADM provides critical insights into pancreatic regeneration and early tumorigenesis and offers a framework for developing targeted strategies for the prevention and treatment of pancreatitis and PDAC.

## Introduction

The pancreas is an important endocrine and exocrine organ. Acinar cells in the exocrine pancreas secrete digestive enzymes zymogens including amylase, trypsin, and lipase, which are released into the duodenum and become activated to digest food [1, 2]. Ductal cells secrete bicarbonate ions to neutralize gastric acid and maintain an optimal pH for enzymatic activity [3, 4]. The endocrine cells secrete hormones such as glucagon and insulin to regulate blood glucose levels [5–8].

Acinar-ductal metaplasia (ADM) is a reversible process in which pancreatic acinar cells dedifferentiate and transdifferentiate into duct-like cells in response to environmental stress such as acute or chronic inflammation, injury, or carcinogenic stimuli [9–12]. This process serves as an adaptive mechanism allowing acinar cells to cope with cell damage or pancreatitis. It is regulated by multiple signaling pathways, including transforming growth factor beta (TGF-β) [13], epidermal growth factor receptor (EGFR) [14, 15], phosphoinositide 3-kinase (PI3K) [16, 17], mitogen-activated protein kinase (MAPK) [18], wingless-related integration site / β-catenin (Wnt/β-catenin) [19, 20], Notch [21], janus kinase-signal transducer and activator of transcription 3 (JAK-STAT3) [22, 23].These pathways control ADM by regulating dedifferentiation, transdifferentiation, proliferation, and cell survival. In addition, immune cell infiltration, release of inflammatory cytokines, and extracellular matrix remodeling significantly influence the formation and maintenance of ADM.

ADM is generally reversible: when the injurious stimulus is removed, acinar cells can restore their original structure and function, and restore the normal physiological state of the pancreas [24]. However, persistent environmental pressure, especially in the presence of genetic mutations such as KRAS mutations, can drive ADM progression to pancreatic intraepithelial neoplasia (PanIN) and pancreatic ductal adenocarcinoma (PDAC), highlighting ADM as a critical precursor to pancreatic cancer [25]. PDAC is among the most prevalent types of pancreatic cancer and exhibits one of the highest mortality rates among all malignant tumors [26]. Approximately 51,980 people die from PDAC per year in the US with the 5-year survival rate of 13% [27]. PDAC is predicted to become the second leading cause of cancer-related deaths in the US by 2030 [28, 29]. Therefore, a comprehensive understanding of ADM formation and its associated with pancreatitis and PDAC is essential for elucidating pancreatic disease pathogenesis.

In this review, we summarize the signaling pathways regulating ADM, the effects of inflammation on ADM and reversal, the progression of ADM to PDAC, heterogeneity of ADM revealed by single-cell studies and potential drugs that prevent ADM.

## Signal pathways in ADM

**Fig. 1:**
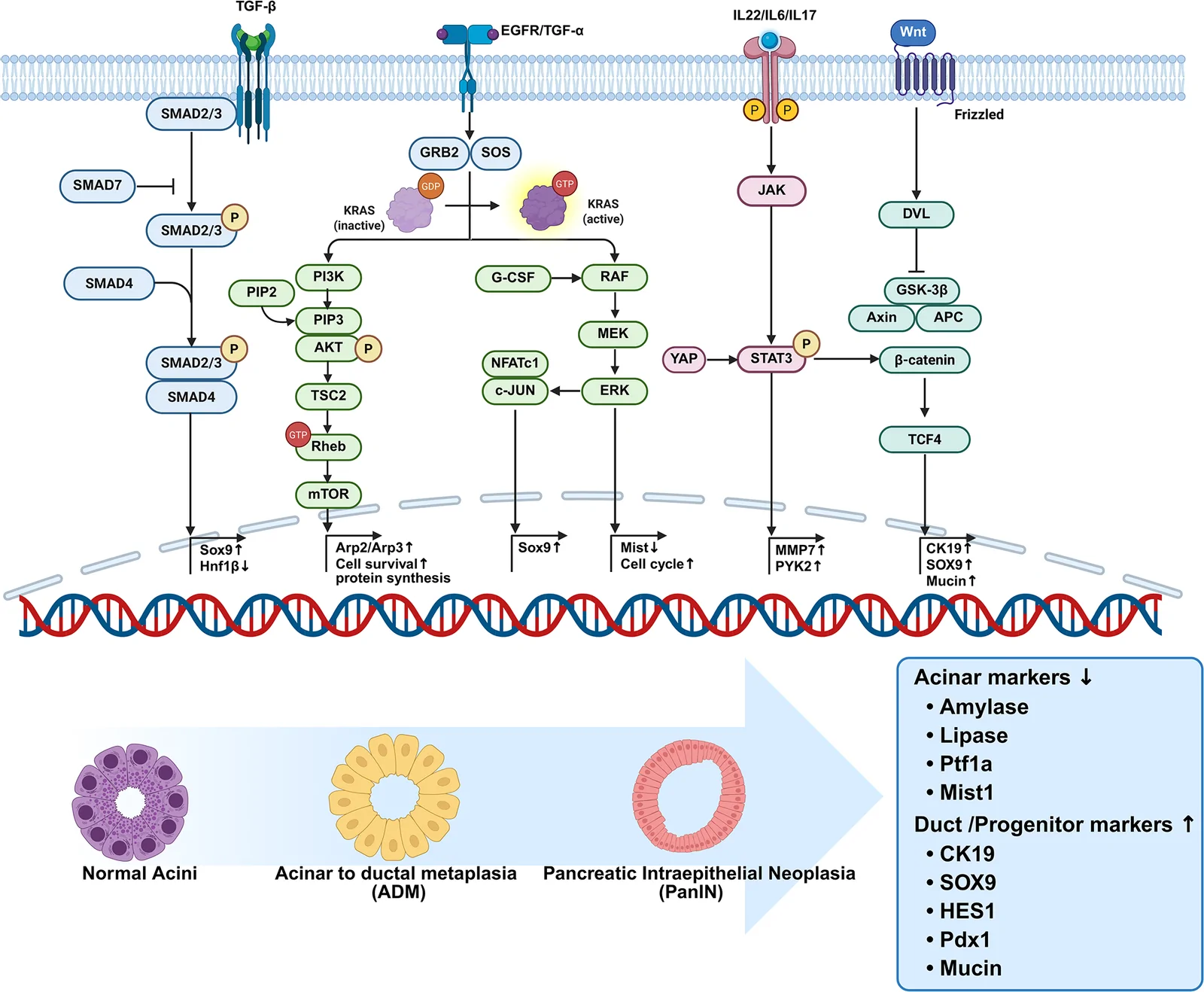
The key signaling pathways in ADM and PanIN formation. TGF-β/SMAD, EGFR–KRAS–RAF–MEK–ERK, PI3K–AKT–mTOR, JAK/STAT, Want/β-catenin The Hedgehog pathway activates downstream transcription factors (such as SMAD, ERK, STAT3, TCF4, etc.) through membrane receptors, jointly regulating cell survival, protein synthesis, cell cycle, and fate determination, promoting the dedifferentiation of acinar cells and obtaining ductal/progenitor cell characteristics. The morphological changes of normal acinar cells progressing from ADM to PanIN are illustrated below. On the right side, changes in molecular markers are listed: acinar markers (Amylase, Lipase, Ptf1a, Mist1) are downregulated, while ductal/progenitor cell markers (CK19, SOX9, HES1, Pdx1, Mucin) are upregulated

Morphologically, ADM is characterized by acinar cells acquiring duct-like features, the most direct indicator of this process [30]. ADM involves dedifferentiation and transdifferentiation: acinar-specific markers(amylase, lipase, carboxypeptidase, ribonuclease) are downregulated, while ductal markers including cytokeratin 19 (CK19), CD133, Cystic fibrosis transmembrane conductance regulator (CFTR) and progenitor markers including Nestin, SRY-box transcription factor 9 (Sox9), Hairy and enhancer of split-1 (Hes1) are upregulated, accompanied by activation of inflammatory factors and other signal pathways [30–32]. Sox9 is absent in normal acinar cells but is expressed in chronic pancreatitis, ADM, PanIN and PDAC, marking its role in acinar reprogramming and cancer development [14, 33, 34]. Figure 1 shows important signal pathways in ADM.

TGF-β regulates pancreatic cell fate, maintaining islet cells, acinar cells, and ductal cells [13, 35]. TGF-β binds to TGF-β RII on the cell membrane, recruiting TGF-β RI and phosphorylating SMAD2/3, which complex with SMAD4 and translocate to the nucleus to regulate transcription factors such as Hnf1β and Sox9, promoting acinar cell dedifferentiation and ADM formation. TGF-β induces ADM in vitro, and co-cultured ductal cells secrete TGF-β to stimulate spontaneous acinar trans-differentiation via SMAD pathway [36, 37]. Inactivation of TGF-β RII or overexpression of SMAD7 can respectively promote or inhibit ADM and pancreatic fibrosis [38, 39].

EGFR and its ligands (EGF and TGF-α) are upregulated during ADM and PanIN regions of Kras^G12D^ mouse model [40, 41]. Ligand binding induces EGFR dimerization and tyrosine phosphorylation, activating downstream proteins. It can activate GRB2, whose SH2 domain bind to phosphorylated EGFR and SH3 domain bind to proline-rich SOS, which in turn can activate KRAS by inducing the exchange of GDP to GTP [42, 43]. With KRAS activation, EGFR can activate pathways including PI3K-AKT-mTOR, JAK-STAT3, RAS-RAF-MEK-ERK [44, 45]. These complex regulatory networks promote cell survival, proliferation, and cytokine release and transdifferentiation. In addition, EGFR can induce the expression of NFATc1 and NFATc4 in acinar cells, and promote the formation of NFATc1 and c-JUN complexes, leading to Sox9 activation and ADM formation [14, 46].

PI3K is a downstream factors of KRAS, which is highly correlated with cell survival and proliferation [47]. PI3K activation converts PIP2 to PIP3, which binds AKT/PKB PH domains, enhancing kinase activity. AKT can further phosphorylate TSC2, preventing negative effect of TSC1/TSC2 complex on Rheb GTPase activity.And Rheb can stimulate mammalian target of rapamycin (mTOR), increasing P70 S6 kinase activity and promoting cell survival, metabolism, and protein synthesis, contributing to ADM [48]. mTOR1/2 remodel the actin cytoskeleton via Arp2/3 complex, causing the transformation of acinar cell morphology [49]. PI3K synergizes with Hippo signaling to upregulate CTGF and Sox9, further promoting ADM [50].

MAPK pathway is another downstream pathway of KRAS, which includes RAF- mitogen-activated protein kinase (MEK)-extracellular signal-regulated kinase (ERK) cascade [18]. RAF activation by KRAS can phosphorylate MEK and ERK and pERK translocate to the nucleus and promotes cell cycle progression. MEK inhibition upregulates Mist1, restoring acinar identity and causing lesion regression, highlighting MAPK’s role in ADM maintenance [51]. MAPK activation also induces upregulation of NFATc1/c-Jun complex, cooperating with JNK to activate Sox9, thus promoting ADM formation [14, 46].G-CSF can activate FRA1 through the MEK/ERK pathway enhancing ADM while reducing immune cells, especially macrophages and neutrophils [52].

Wnt/β-catenin pathway is critical for ADM. β-catenin stabilization and nuclear translocation activate target genes. The ectopic activation of hedgehog in ductal cells signaling can enhance Wnt/β-catenin activity via TCF4, promoting ADM [19, 20]. The role of the Wnt/β-catenin pathway in ADM depends on the progress of cell reprogramming. In pancreatitis, this pathway can be quickly activated within one week and can block the production of ADM induced by KRAS [53]. However, one month later, accumulation of β-catenin was observed in acinar cells expressing Kras^G12D^, CK19 and mucin. In contrast, cells lacking β-catenin did not exhibit upregulation of CK19, indicating that this pathway can promote ADM at this time [20]. Sirt-1mainly translocates from the nucleus to the cytoplasm in ADM and regulates the activity of Wnt/β-catenin and PTF1a through deacetylation, thus supporting cell viability and promoting ADM formation [54]. Furthermore, Wnt pathway activation can enhance MAPK signaling, synergistically promoting ADM development.

Notch signaling (ligands: Jagged-1; receptors: Notch2/3; targets: Hes1, Hey1) is upregulated in ADM [21, 55]. This pathway can be activated via TGF-α and KRAS through ADAMs, MMPs and PKD1, and further releasing NICD, which regulates Hes1 [56, 57]. Hes-1 can negatively regulate Mist1, affecting digestive enzyme production, exocytosis and acinar homeostasis, thereby promoting ADM [58, 59].

JAK-STAT3 signaling is activated by G-protein-associated receptors, YAP1/TAZ, IL22, inducing genes controlling proliferation, apoptosis, and inflammatory cytokines [23, 60, 61].STAT3 can increase MMP7 expression, facilitating ECM remodeling and ADM formation [22]. In addition, YAP/TAZ activated STAT3 can further stimulate PYK2 and Wnt/ β- catenin pathways, reinforcing [62].

Recent studies have demonstrated that epigenetic reprogramming plays a crucial role in initiation and progression of ADM. During ADM formation, acinar cell identity genes such as Ptf1a and Mist1 undergo epigenetic silencing, accompanied by chromatin remodeling and histone modifications that promote ductal-like transcriptional programs [17, 33, 63]. Polycomb repressive complexes, particularly EZH2-mediated H3K27 trimethylation, contribute to the epigenetic reprogramming during ADM, whereas disruption of SWI/SNF chromatin-remodeling complexes, including ARID1A, facilitates the loss of acinar differentiation and promotes stabilization of metaplastic states [64, 65]. DNA methylation changes have also been identified during ADM transition, particularly in genes associated with KRAS downstream signaling, suggesting the existence of an “epigenetic memory” that may predispose metaplastic cells to persistent pre-neoplastic transformation [17]. In addition, non-coding RNAs, especially miRNAs such as miR-802, have been shown to regulate ADM formation by modulating cytoskeletal organization, Sox9 activity, and acinar cell plasticity [66].

In summary, ADM is driven by coordinated signaling networks including TGF-β, EGFR, PI3K, MAPK, Notch, Wnt/β-catenin and JAK/STAT3.These pathways promote acinar dedifferentiation, fibrosis, and inflammation, contributing to chronic pancreatitis and increasing PDAC risk. Understanding pathway interactions is essential for identifying therapeutic targets for preventing ADM and its progression to cancer.

## ADM in pancreatitis

### Mechanisms of ADM formation in acute pancreatitis

**Fig. 2:**
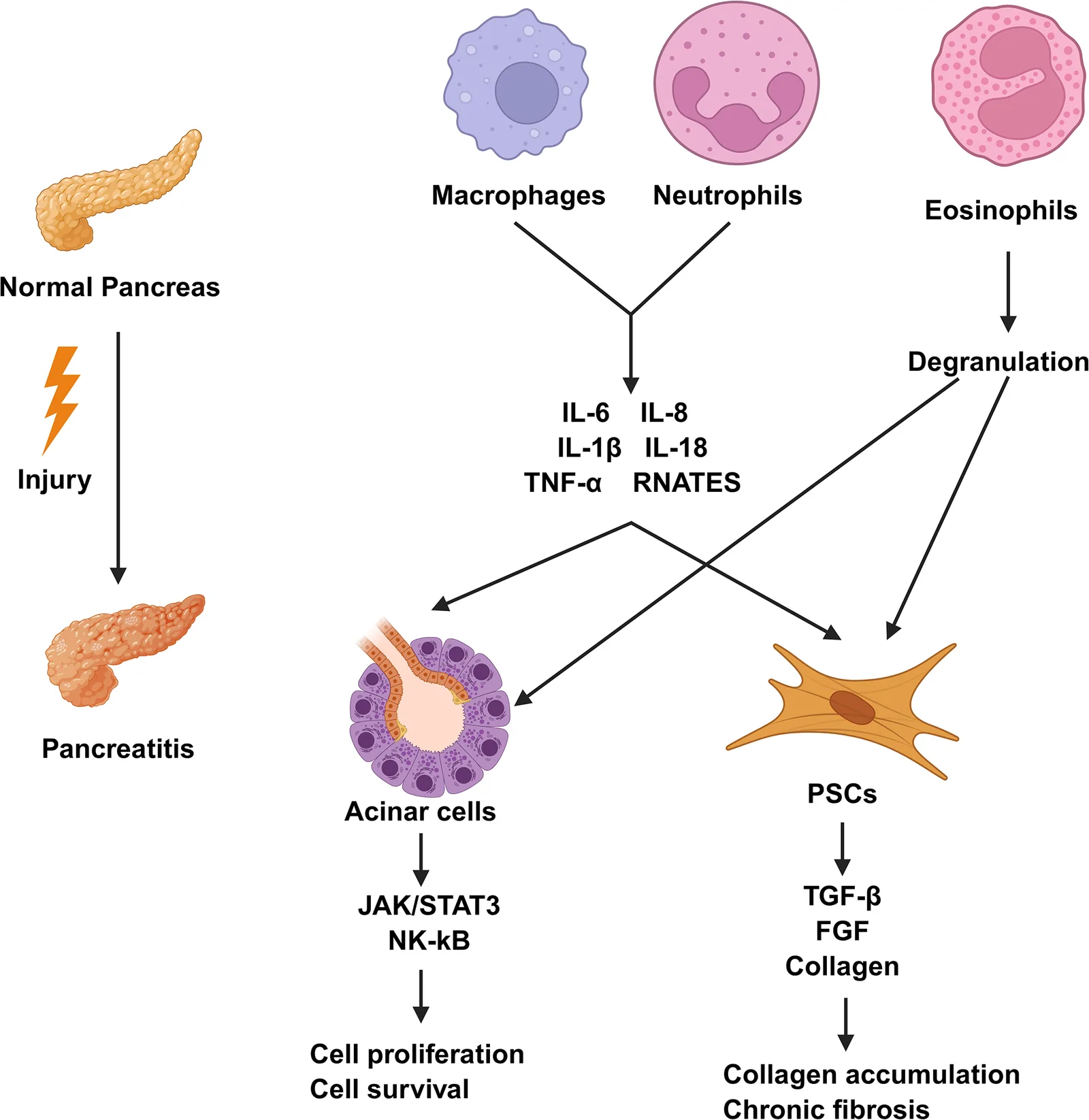
Activation of immune cells in ADM. After pancreatic injury, pancreatitis is induced, and macrophages and neutrophils release inflammatory factors such as IL-6, IL-1 β, TNF - α, IL-8, etc. Eosinophils participate in the inflammatory response through degranulation. These signals work together on acinar cells and PSCs, activating the JAK/STAT3 and NF-κB pathways to promote cell survival and proliferation, as well as inducing TGF-β, FGF, and collagen secretion, leading to collagen deposition and chronic fibrosis

Pancreatitis is classified as acute and chronic pancreatitis. Acute pancreatitis involves acinar cell injury and impaired exocrine function, initiating a strong inflammatory response in the pancreas [67]. This inflammatory environment promotes the dedifferentiation of acinar cells into progenitor-like cells, facilitating tissue repair through differentiation, proliferation, and remodeling. Normally, once the acute inflammation resolves, acinar cells will generally return to their original state and resume the secretion of digestive enzymes, as transient inflammatory signaling (e.g., TNF‑α, IL‑6, NF‑κB) induces reversible acinar plasticity [68]. In the experimental models of acute pancreatitis (e.g., caerulein), ADM is typically reversible, and pancreatic morphology and function are stored, reflecting the inherent plasticity of acinar cells [69]. The mechanisms of ADM in pancreatitis are shown in Fig. 2.

### Mechanisms of ADM formation in chronic pancreatitis

Persistent inflammatory stimuli induce PSCs remodeling and sustained cytokine signaling, leading to irreversible pancreatic fibrosis and chronic pancreatitis. Under these conditions, acinar cells maintain a duct-like morphology and function, leading to ADM formation. Chronic pancreatitis with pancreatic fibrosis is strongly associated with pancreatic cancer and represents an independent risk factor, increasing the incidence of pancreatic cancer 20‑50 fold [70].

A hallmark of chronic pancreatitis is the excessive extracellular matrix deposition accompanied by infiltration of macrophages and fibro‑inflammatory cells. Pancreatic injury activates acinar cells, macrophages, and neutrophils, inducing the production of pro‑inflammatory cytokines such as IL‑6 via the JAK/STAT3 pathway and IL‑8 via the MAPK/ERK pathway, as well as various chemokines and growth factors, thereby activating quiescent PSCs [71]. Long‑term activation of these signaling pathways has been linked to ADM stabilization and inhibition of reversal observed in acute injury, contributing to persistent metaplasia in chronic pancreatitis [36].Necrosis of acini and infiltration by inflammatory cells lead to secretion of high levels of inflammatory factors such as IL‑1β and TNF‑α, which activate TGF‑β and FGF in PSCs via paracrine secretion, promoting pancreatic fibrosis and ADM during chronic pancreatitis [72, 73]. Macrophages are central to chronic pancreatitis and ADM formation through secretion of inflammatory mediators [74, 75]. They secrete RANTES and TNF‑α, activating NF‑κB signaling pathways in acinar cells and its downstream genes involved in cell proliferation, cell survival, and extracellular matrix remodeling, thus inducing ADM formation [76]. In addition, IL‑18 secreted by macrophages is also a key factors driver of ADM. Accumulated macrophages activate IL‑18 via NLRP3 inflammasome and promote chronic eosinophilic inflammation, potentially serving as an intermediate step from chronic pancreatitis to PDAC [77]. Eosinophil degranulation induces the TGF‑β/SMAD4 pathway, stimulating EMT and PSCs activation. The eosinophil granule protein EPX upregulates TGF‑β, KRAS, and MUC2, resulting in collagen accumulation and ADM formation [78].

In vitro studies indicate that various inflammatory factors such as TNF‑α and IL‑17 increase IL‑6 secretion by peripancreatic myofibroblasts and macrophages, supporting the role of IL‑6 in pancreatitis [79]. IL‑6 is a critical pro‑inflammatory cytokine, elevated in serum of patients with acute and chronic pancreatitis, correlating with inflammation severity [80, 81]. IL‑6 binds to the IL‑6R and gp130 to form a signaling complex, activating JAK kinase and promoting STAT3 phosphorylation, leading to ADM formation [68]. Finally, long‑term chronic pancreatitis leads to sustained activation of pathways (e.g., Notch, EGFR/MAPK) consolidating ADM and prevent reversal, resulting in a persistent duct‑like state that increases PanIN and PDAC risk.

### The significance of ADM in the inflammatory environment

ADM demonstrates the high plasticity of acinar cells, potentially serving as an intrinsic defense mechanism of the host [82]. Acinar cells are particularly sensitive to external stimuli compared with other pancreatic cell lineages and can maintain tissue stability via metaplasia under genetic or environmental stress [83]. They store abundant proteases and amylases, and cellular damage can result in enzyme release, harming the surrounding cells and tissues. Duct-like cells exhibit greater resistance to digestive enzymes than acinar cells. ADM in acute pancreatitis is generally considered a transient adaptive and regenerative response that protects tissue from autodigestive injury by temporarily suppressing the highly secretory acinar phenotype. Inflammatory signaling is short-lived and metaplastic cells retain plasticity, allowing re-differentiation into mature acinar cells after resolution of injury [51, 84]. In contrast, chronic inflammatory conditions promote stabilization of ADM and progression toward persistent duct-like and pre-neoplastic states. It is characterized by sustained activation of inflammatory and oncogenic pathways, including STAT3, NF-κB, EGFR/MAPK, and Notch signaling, which reinforce duct-like identity and inhibit acinar re-differentiation. In addition, fibrosis-associated stromal remodeling and continuous cytokine exposure create a tumor-promoting microenvironment that facilitates the transition from ADM to PanIN and eventually PDAC. Recent studies further demonstrate that, compared with the regenerative ADM observed in acute injury, chronic inflammation-associated ADM exhibits transcriptional programs enriched for fibrosis, cytokine signaling, and oncogenic pathways, thereby increasing susceptibility to pancreatic tumorigenesis.

Although the mechanisms by which ADM supports regeneration or contributes to pancreatic tumorigenesis remain unclear, multiple signaling pathways are involved. Therefore, understanding ADM reversibility and the key regulatory factors is critical for insights into pancreatic regeneration and tumorigenesis.

### The recovery mechanism of ADM after inflammation

Once the inflammatory stimulus resolves, acinar cells can restore their mature phenotype and rebuild normal acinar structure and function through coordinated regulatory networks. ADM reversal involves reactivation of acinar-specific transcription networks, rather than simple cell death [85]. This reversible process is facilitated by dedifferentiation followed by re-expression of acinar-specific transcription factors including Mist-1 and PTF1A, which support acinar maturation and functional recovery. Sox4 inhibits dedifferentiation and helps maintain acinar identity during ADM reversal, highlighting the role of endogenous molecules in preserving and restoring acinar maturity after inflammation [10].

After inflammation, the pancreatic extracellular matrix undergoes remodeling. As the inflammation resolves, normal extracellular matrix structure gradually recovers, which is crucial for directed cell migration, proliferation, and differentiation. Matrix metalloproteinases (MMP), especially MMP2 and MMP9, facilitate restoration of normal pancreatic tissue structure and promote reconstruction of acinar and ductal systems by degrading abnormally accumulated extracellular matrix components [86]. Although acinar cell morphology is restored after inflammation, cells do not fully recover to their original state, retaining epigenetic modifications, including altered chromatin accessibility and histone marks such as H3K4me1. This inflammatory memory can influence acinar cell response to KRAS mutations, increasing susceptibility to carcinogenesis [87].

### Other environment factors promoting ADM

**Fig. 3:**
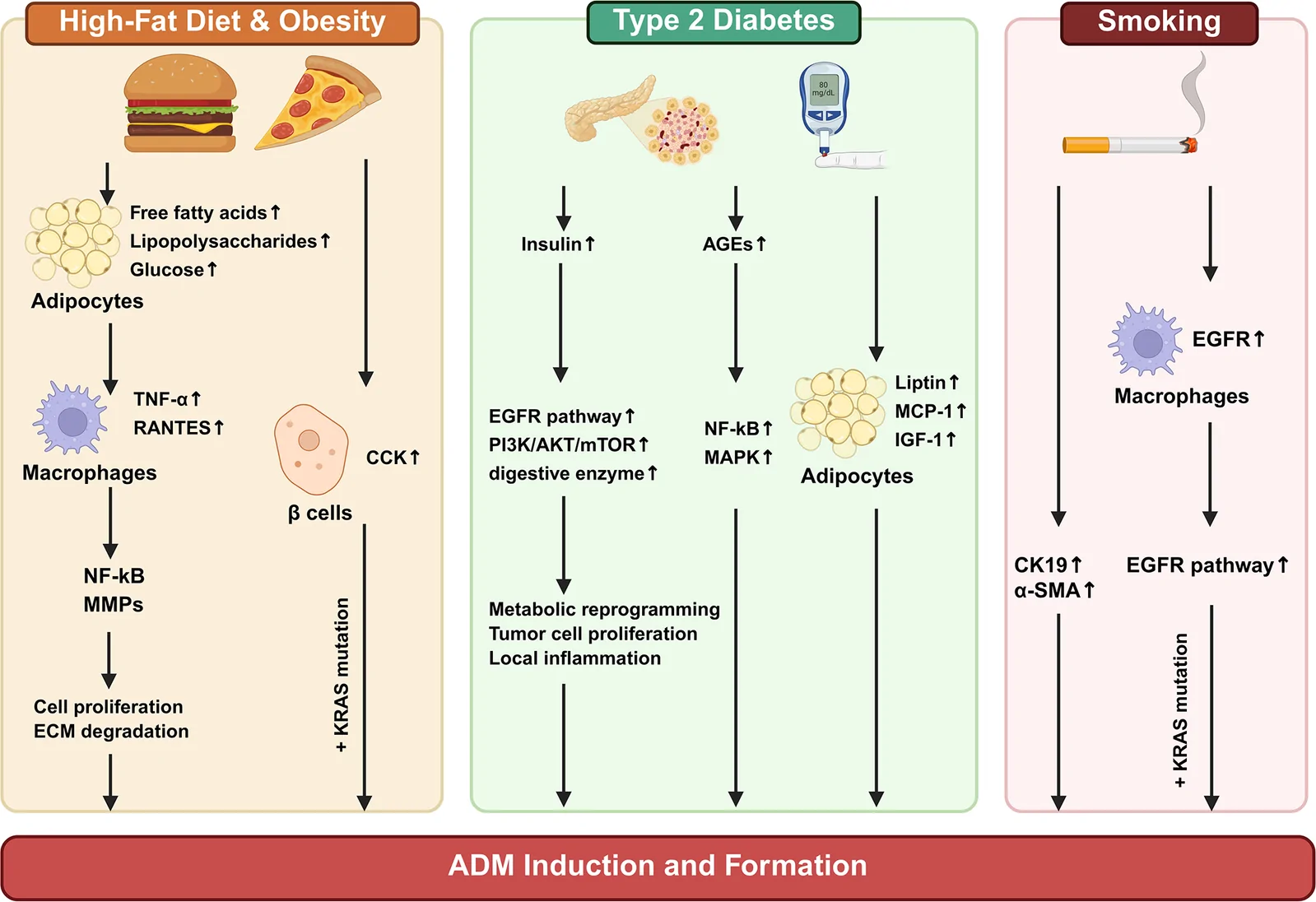
The influence of environmental factors and lifestyle on the formation of ADM. High fat diet and obesity activate macrophages through lipid metabolism disorders and chronic inflammation, promoting the activation of inflammatory factors such as TNF-α and RANTES, as well as pathways such as NF-κ B and MMPs, thereby enhancing ADM formation. Diabetes related hyperglycemia, hyperinsulinemia and AGEs accumulation can drive metabolic reprogramming and local inflammation through AKT/mTOR, MAPK, NF - κ B and EGFR signaling pathways, and promote the occurrence of ADM. Smoking can further promote the formation of ADM and increase the risk of pancreatic cancer by up regulating CK19, α - SMA and EGFR, and cooperating with KRAS mutation

In addition to genetic factors, environmental factors, and lifestyle habits can influence ADM formation. Figure 3 shows the impact of environmental factors on ADM.

High-fat diet and obesity increase ADM risk. Diet-induced obesity in rats enhances ADM prevalence, but this effect can be reversed after weight loss surgery, indicating that obesity induced ADM is reversible [88]. Lipid metabolism affects inflammation and immune responses. Obesity leads to enlarged adipocytes and metabolic by-products, including free fatty acids, lipopolysaccharides, and glucose, activating pro-inflammatory macrophages, which further secrete TNF- α, and RANTES, further activating NF-κB and MMPs in ADM cells, thereby promoting ADM formation [76, 89]. MMP9, a novel protease-activated receptor 1 agonist, activates the Myc pathway and locks cells in a duct-like cell state [90, 91]. Obesity may also stimulate aberrant islet β cells secreting cholecystokinin (CCK) with unknown reasons, promoting ADM formation in the context of KRAS mutation [92]. Alcohol intake and obesity can activate autophagy via IL-6 and TNF-α, degrading damaged proteins to enhance adaptability, ultimately contributing to ADM formation [93].

Type II diabetes is another important risk factor for PDAC [94]. New-onset diabetes has a higher risk of inducing PDAC than long-term diabetes [95]. Hyperglycemia provides more energy for cancer cells via enhanced anaerobic glycolysis [96]. Hyperglycemia can also induce EGF and EGFR to promote the proliferation of tumor cells [15]. Hyperinsulinemia and IGF activate the AKT/mTOR pathway, inducing metabolic reprogramming, especially glucose and lipid metabolism [97].The accumulation of advanced glycation end products (AGEs) can bind to its receptors and activate downstream tumor promoting signals, such as NF-κB and MAPK [98]. Hyperinsulinemia promotes digestive enzyme secretion in exocrine cells through insulin receptors, leading to local inflammation and tissue damage. Insulin also activates PI3K/AKT/mTOR and MAPK/ERK pathways, jointly promoting ADM formation [99]. Accumulated adipocytes in the pancreas secrete leptin, MCP-1 and IGF-1, further promoting ADM formation [100]. Short-term metabolic reprogramming and inflammatory changes may be reversible, but long-term activation of these pathways stabilizes ADM, potentially progressing to PanIN and PDAC.

Smoking is another well-established risk factors for PDAC [101, 102]. Mechanistically, cell model and mouse model confirm that tobacco-specific nitrosamines such as nitrosamine 4-(methyl nitrosamine)-1-(3-pyridyl)-1-butanone (NNK) induce cyclic AMP response element-binding protein (CREB) phosphorylation via granulocyte-macrophage colony-Stimulating factor (GM-CSF) signaling, promoting inflammatory responses and Akt activation, enhancing proliferation and survival of pancreatic ductal cells [103]. Chronic exposure to smoking inhibits autophagy and apoptosis through ROS-dependent Akt activation, creating a pro-survival environment for neoplastic transformation [104]. Smoking also alters epigenetic regulation: HDAC3 upregulation in pancreatic cells increases IL-6 secretion and M2 macrophage polarization, remodeling the tumor microenvironment and facilitating ADM formation [105]. Smoke exposure also promotes CK19 and α-SMA expression, and increases HB-EGFR derived from tumor tumor-associated macrophages (TAMs), combined with KRAS mutation promoting ADM formation [106]. While reversibility of smoking-induced ADM is unclear, removal of smoking may allow partial recovery of the inflammatory and immune microenvironment.

## Acinar-ductal metaplasia in pancreatic cancer

PanIN is the most common precancerous lesion of pancreatic cancer, classified into low-grade PanIN and high-grade PanIN based on cellular atypia [107]. Persistent KRAS mutations in ADM cells drive progression to PanIN. This transformation involves complex molecular events, including changes in cellular function and interactions with the microenvironment. KRAS mutations result in constitutive activation of RAS proteins by maintaining GTP binding status, independent of external stimuli. This persistent activation of downstream RAF/MEK/ERK and PI3K/AKT pathways enhances proliferation and migration, inhibits apoptosis, and promotes ADM progression to PanIN.

During ADM progression to PanIN, ductal markers such as Sox9 are upregulated, whereas acinar markers including PTF1a and Mist1 are downregulated. PTF1a deficiency enhances acinar cell sensitivity to KRAS mutations, thereby accelerating tumorigenesis [63]. While PTF1a overexpression in PDAC cells reduces proliferation and inhibits ABC transporter activity, slowing tumor progression [108]. Mist1 maintains acinar structure and function, whose loss activates EGFR and Notch pathways, promotes PanIN formation and increases proliferative capacity and morphological alterations [109]. Additionally, loss of Mist1 also disrupts cell polarity and gap junctions, enhancing susceptibility to dedifferentiation and carcinogenic stimuli [110]. Although Sox9 is not essential for ADM, it facilitates PanIN formation in the context of KRAS mutations. Upstream factors including EGFR/NFATc1 and BRG1 induce Sox9 expression via multiple pathways, promoting lineage conversion [14, 33, 111].

Additional genes are also crucial in KRAS-driven pancreatic tumorigenesis. ATF3 acts as a key cofactor, promoting PanIN-to-PDAC progression by inhibiting DUSP5/DUSP6 and reducing ERK phosphorylation [112]. KRAS mutations also upregulate CIP2A, which suppresses PP2A-B56α activity, leading to MYC stabilization and accumulation, thereby driving carcinogenic [113]. Hes1 is a critical regulator throughout KRAS-induced ADM to PanIN and to PDAC progression. It is continuously expressed from ADM onset, and knockout studies in mice show that ADM reverts to normal acinar cells instead of PanIN when Hes1 is absent [114]. However, if PanIN has already occurred, Hes1 primarily functions to inhibit the progression of PanIN and PDAC. The absence of Hes1 enhances the epithelial-mesenchymal transition through the upregulation of Muc5ac during PDAC progression. In summary, Hes1 promotes the progression from ADM to PanIN but inhibits the further development of PanIN and PDAC [115].

Progression from low-grade PanIN to high-grade PanIN and to PDAC is driven by the subsequential acquisition of genetic alterations, including CDKN2A (p16) inactivation, TP53 mutation or loss, and SMAD4 deletion. These alterations compromise cell cycle regulation, DNA damage response, and TGF-β signaling, enabling malignant clonal expansion. Recent analysis of human samples and genetically engineered mouse models (GEMMs) continue to support this pathway [116].

In addition to KRAS‑driven and inflammation‑induced ADM, KRAS‑independent mechanisms also contribute to ADM in PDAC. Metaplastic acinar cells undergo genetic reprogramming, resulting in DNA methylation of genes such as PI3K and Rho/Rac/Cdc42 alongside transcriptional activation. Importantly, recent epigenomic analyses demonstrate that transcription factors like KLF4 can initiate ADM and establish a DNA methylation ‘memory’ of the transition state even in the absence of oncogenic KRAS, indicating that epigenetic reprogramming constitutes another KRAS‑independent route to ADM formation [17]. However, when the damage is repaired, this DNA methylation will still be retained, which may have a potential role in the development of pancreatic cancer. Transforming growth factor‑β1 (TGF‑β1) can directly induce ADM through SMAD‑dependent signaling, without requiring KRAS mutations, highlighting growth factor pathways as alternative drivers [36].

Beyond genetic alterations, the tumor microenvironment (TME) critically influences ADM progression to PanIN and PDAC [117, 118]. Persistent inflammation, activated PSCs, immune infiltration, and ECM remodeling create a niche that supports neoplastic transformation [119, 120]. Cytokines and growth factors, including IL-6, TNF-α, TGF-β, CXCLs, and HB-EGF, activate STAT3, NF-κB, MAPK, and TGF-β pathways in metaplastic epithelial cells [13, 61, 121]. PSCs and CAFs drive collagen deposition and desmoplasia, stabilizing ADM and promoting PanIN progression [122, 123]. Stromal–epithelial interactions further suppress immunity via MDSCs, regulatory T cells, and alternatively activated macrophages, facilitating malignancy [124]. Emerging spatial and single-cell studies suggest these inflammatory and fibrotic niches act as early precursors of the PDAC microenvironment, actively driving the ADM–PanIN–PDAC sequence [125].

## Heterogeneity of ADM revealed by single-cell studies

Recent advances in single-cell RNA sequencing (scRNA-seq) have revealed that ADM is not a homogeneous cell population but a complex mixture of transcriptionally distinct cell states. During pancreatic injury or chronic stress, acinar cells first adopt a mucin/ductal progenitor state, reminiscent of gastric pyloric-type metaplasia, expressing markers such as Tff2 and Muc6 [125]. From this transitional progenitor, multiple lineages emerge including tuft cells, rare chemosensory cells expressing Pou2f3 and Dclk1, and enteroendocrine cell (EEC) subtypes producing hormones like serotonin, ghrelin, and somatostatin [125, 126]. Additional ADM subpopulations include gastric pit- and chief-like cells, proliferative, and senescent subclusters, indicating that ADM is a spectrum of cell states rather than a unidirectional ductal fate [126]. Lineage trajectory inference and RNA velocity analyses indicate that these heterogeneous ADM subpopulations follow distinct differentiation paths. Some transitional progenitors can revert toward acinar identity under resolving injury, suggesting plasticity in reversible ADM, whereas other subtypes—particularly gastric pit- and chief-like clusters expressing oncogenic transcription factors such as Onecut2 and Foxq1—show a predilection for pre-neoplastic transformation and persistent ADM in the context of Kras activation [121, 126]. These observations highlight that the cellular heterogeneity and lineage trajectories of ADM are critical for determining whether metaplasia resolves or progresses toward PanIN and PDAC. Notably, the identification of transcriptionally distinct ADM subpopulations associated with oncogenic signaling and persistent metaplastic states suggests that these cell populations may represent candidate biomarkers or potential therapeutic targets for early pancreatic tumorigenesis. In particular, markers such as Dclk1, Onecut2, and Foxq1 identified in high-risk ADM clusters may help to distinguish persistent or pre-neoplastic ADM states from reversible injury-associated metaplasia, although their clinical utility in early PDAC detection and targeted therapy remains to be validated in human studies. Understanding the molecular and cellular diversity of ADM provides insight into the mechanisms underlying pancreatic regeneration and early tumorigenesis.

## Drugs to prevent ADM formation

Due to the critical role of ADM formation in the pathogenesis of pancreatitis and PDAC, multiple potential drugs targeting ADM-associated pathways are under investigation, which are shown in Table 1.

**Table 1:** Drugs to prevent ADM formation and their mechanisms

Drugs	Mechanism	Key findings	References
Trichostatin A	Pan-HDAC inhibitor; downregulates Sirt1	Reduces & reverses ADM in mouse organoids via Spink1/PI3K/AKT	[85, 127]
MS-275	Type I HDAC inhibitor; downregulates EGFR	Inhibits TGF-α-driven ADM cell-autonomously	[128]
LLL12B	JAK/STAT3 inhibitor (blocks p-STAT3)	Blocks ADM; reverses only in non-KRAS mutant models	[85]
Metformin	STAT3 signaling inhibitor	Reduces ADM area and delays tumorigenesis in KRAS mutant/CP mouse models	[129, 130]
Ibuprofen	NSAID; reduces M1 cytokines (TNFα, RANTES)	Attenuates pancreatitis-associated ADM via NF-κB suppression	[76, 131]
Baicalein	NF-κB inhibitor; reduces TNFα/NO	Suppresses ADM in vitro by inhibiting acinar NF-κB and macrophage inflammation	[132, 133]

Trichostatin A (TSA), a pan histone deacetylation (HDAC) inhibitor, can downregulate Sirtuin-1 (Sirt1) at the transcript level and modulates ADM by deacetylating PTF1a and β-catenin [127]. In organoid cultures derived from p48^cre/+^ and KC mice, TSA reduces the proportion of ADM areas and CK19 expression, while doubling the expression of acinar-specific genes. TSA also reverses existing ADM, restoring acinar-like morphology in a concentration dependent manner through SRINK1 and PI3K/AKT signaling, indicating its capacity to both block and reverse ADM in vitro [85].

MS-275, a type I HDAC inhibitor, decreases ADM formation induced by TGF-α mediated EGFR activation. It inhibits EGFR and its ligand and downregulates ADM markers such as CK19 and Sox9 and maintains metabolic activity in non-ADM cells, suggesting that ADM reduction occurs via cell-autonomous mechanisms rather than diminished external stimuli [128].

The JAK/STAT3 pathway contributes to ADM formation. Treatment with LLL12B reduces STAT3 phosphorylation, thereby inhibiting JAK/STAT3 pathway. In organoid cultures derived from p48^cre/+^ and KC mice, LLL12B decreases CK19 expression and ADM formation. It also increases the expression of acinar markers (AMY2A, CPA2, CELA1) in p48^cre/+^ organoids, but not in KC organoids, indicating that LLL12B can reverse ADM only in absence of KRAS mutations, while only partially inhibiting ADM in the presence of KRAS mutation [85]. The precise mechanism remains to be clarified.

Metformin, a widely used drug for type II diabetes, has demonstrated potential anti-tumor effects, including cytotoxicity, immune regulation, mTOR inhibition [129]. In KRAS-mutant and chronic pancreatitis mouse models, metformin reduces ADM areas and delays tumor formation, potentially via inhibition of STAT3 pathways. In other cancer types, metformin suppresses JAK/STAT3/c-MYC signaling in esophageal squamous cell carcinoma and COX2/PEG2/STAT3 signaling in prostate cancer, although the precise mechanisms in ADM and PDAC remain unclear [130].

Ibuprofen, a non-steroidal anti-inflammatory drug used to alleviate pancreatitis-associated pain, selectively suppresses M1 macrophage activation and reduces pro-inflammatory cytokines such as TNFα and RANTES [131]. Since TNFα and RANTES activate NF-κB and downstream targets in acinar cells to induce ADM, ibuprofen can also reduce ADM formation after acute pancreatitis [76].

Baicalein, a flavonoid from Scutellaria baicalensis Georgi, traditionally used to treat pancreatitis in China [133], inhibits NF-κB activation induced by TNF-α in AR42J rat acinar cells, suppresses TNF-α and nitric oxide (NO) secretion by macrophages, and reduces LPS-induced inflammatory responses, collectively attenuating ADM formation in vitro [132].

However, there are no published human clinical trials or direct clinical research studies on and of the compounds above for treating or preventing ADM. There are several reasons. First, reliable methods for the early identification and dynamic monitoring of ADM in humans are currently lacking. ADM lesions are typically microscopic and diffusely distributed, making them difficult to detect using imaging modalities or blood-based biomarkers, while no biomarkers or diagnostic criteria have yet been established. In addition, ADM itself exhibits a certain degree of reversibility and may represent a physiological tissue repair response rather than an obligate precancerous lesion. Therefore, the clinical benefit of therapeutically targeting ADM remains uncertain, as not all ADM lesions inevitably progress to pancreatic cancer.

## Discussion

ADM is a crucial process in the pancreas, representing a cellular adaptation in response to environmental and inflammatory stimuli. While it is reversible under acute conditions, persistent inflammation or genetic mutations, particularly KRAS mutations, can drive the progression of ADM towards PanIN and ultimately PDAC. Various signaling pathways, including TGF-β, EGFR, PI3K, MAPK, and Notch, play pivotal roles in mediating ADM formation and progression, highlighting their potential as therapeutic targets for preventing ADM and its malignant transformation. Additionally, the inflammatory environment, particularly in pancreatitis, significantly influences ADM development, with immune cells and cytokines, such as IL-6, IL-18, and TNF-α, contributing to the pathogenesis of both chronic pancreatitis and pancreatic cancer. Understanding the molecular mechanisms underlying ADM and its progression to PDAC is essential for developing new strategies for early detection and intervention. Furthermore, emerging drugs targeting specific signaling pathways, such as HDAC inhibitors, JAK/STAT3 inhibitors, and anti-inflammatory agents, show promise in preventing ADM formation and potentially halting the progression of pancreatic cancer. Continued research into these pathways and therapies is critical to improving the prognosis for patients at risk of pancreatic cancer.

Despite the deepening understanding of the molecular mechanism of ADM in recent years, there are still several urgent issues that need to be addressed. Firstly, the “promoting” or “inhibiting” roles played by different signaling pathways in different stages of ADM have obvious temporal and situational dependencies, and their precise regulatory mechanisms have not been fully elucidated [33, 74]. ADM research urgently needs to move from “static mechanism description” to “dynamic and systematic analysis”. Single cell transcriptomics, spatial genomics, and organoid models should be utilized to finely characterize the cellular lineage evolution and signaling pathway interactions of ADM at different time points and microenvironments [17, 125, 134–136]. Secondly, how the residual epigenetic “inflammatory memory” during ADM reversal affects subsequent sensitivity to oncogenic mutations still requires systematic research [137, 138]. Further clarification is needed on which molecular events determine the reversible repair or irreversible tumorigenesis of ADM, in order to define the optimal intervention window. In addition, how environmental factors such as inflammation, metabolic abnormalities (such as obesity and diabetes) and smoking work together to shape the cancer promoting microenvironment is also an important entry point to understand the transformation of ADM to PDAC [139].

At the level of treatment and prevention, interventions targeting key pathways of ADM, such as HDAC inhibitors, JAK/STAT3 inhibitors, anti-inflammatory drugs, and metabolic regulators, have shown the potential to block or reverse ADM in experimental models [85]. However, their efficacy differences and long-term safety under different genetic backgrounds, especially KRAS mutation status, still need further validation. Future research should combine single-cell omics, spatial transcriptomics, and organoid models to systematically analyze the dynamic evolution process of ADM and its interaction with the immune microenvironment, in order to screen for more specific and time sensitive intervention targets [125, 140]. In general, in-depth understanding of the dual biological significance of ADM and finding a precise intervention window between “reversible repair” and “irreversible tumorigenesis” will provide new theoretical basis and treatment strategies for early prevention and treatment of pancreatitis and pancreatic cancer [141].

In general, an in-depth understanding of the “double-edged sword” role of ADM between inflammation repair and tumorigenesis will provide a new theoretical basis and targeted direction for the regulation of tissue regeneration of pancreatitis and the early prevention and treatment of pancreatic cancer.

## Data Availability

No datasets were generated or analysed during the current study.
